# Free distribution of insecticidal bed nets improves possession and preferential use by households and is equitable: findings from two cross-sectional surveys in thirteen malaria endemic districts of Bangladesh

**DOI:** 10.1186/1475-2875-10-357

**Published:** 2011-12-13

**Authors:** Syed M Ahmed, Shamim Hossain, Mohammad M Kabir, Sanjit Roy

**Affiliations:** 1 Research and Evaluation Division, BRAC Centre, 75 Mohakhali, Dhaka Dhaka-1212, Bangladesh; 2 BRAC Health Programme, BRAC Centre, 75 Mohakhali, Dhaka Dhaka-1212, Bangladesh

**Keywords:** Malaria, LLINs, ITNs, BRAC, Bangladesh

## Abstract

**Background:**

BRAC, an indigenous non-governmental development organization (NGO), has been implementing a programme to prevent and control malaria in the 13 malaria-endemic districts of Bangladesh since 2007. One of the critical preventive interventions is the distribution of insecticidal bed nets (long-lasting insecticide-treated nets, LLINs and insecticide-treated ordinary nets, ITNs) to the community free of cost. This study aimed to assess progress in the possession, preferential use, and knowledge on use of the LLIN/ITNs including the programme's avowed pro-poor inclination one and three and half years after intervention began.

**Methods:**

A convenient sampling strategy based on malaria endemicity in the districts was adopted. First, thirty *upazila *(sub-district, with a population around 250,000)*s *were selected at random, with high prevalent districts contributing more *upazilas*; second, from each *upazila*, one (2008) to two (2011) villages (covered by insecticidal bed net distribution programme) were selected. From each village, households that had either one under-five child and/or a pregnant woman were included in the survey, one household being included only once. Data were collected using a pre-tested structured questionnaire.

**Results:**

In all, 3,760 households in 2008 and 7,895 households in 2011 were surveyed for collecting relevant information. Proportion of households with at least one LLIN, and at least one LLIN/ITN increased (22-59 to 62-67% and 22-64% to 74-76% respectively) over time, including increase in the mean number of LLIN/ITNs per household (≤ 1 to 1 +). The programme achieved > 80% coverage in sleeping under an LLIN/ITN in the case of under-five children and pregnant women, especially in the high-endemic districts. Knowledge regarding critical time of hanging the net also increased over time (7-22 to 44-54%), but remained low. The pro-poor inclination of the programme is reflected in the status of relevant indicators according to self-rated poverty status of the households.

**Conclusions:**

There has been a substantial improvement in possession and usage of insecticidal bed nets especially for the two most vulnerable groups (under-five children and pregnant women), including a reduction of gaps between the high and low endemic districts, and the deficit and non-deficit households during the study period.

## Background

Malaria is a public health problem in some 90 countries and is estimated to be responsible directly for about one million deaths annually or 3,000 deaths a day world-wide [1,2]. While Africa accounts for 90% of the mortality burden for malaria (mostly at home), South-east Asia accounts for 9% of the burden. Out of 11 countries of the World Health Organization South-east Asia Regional Office (WHO SEARO), 10 countries including Bangladesh are malaria endemic. The 13 eastern districts of Bangladesh are highly endemic with an overall prevalence of 3.1% (*Plasmodium falciparum *2.73%, *Plasmodium vivax *0.16% and mixed infection 0.19% by rapid diagnostic test 'FalciVax'), with highest prevalence being in the three Chittagong Hill Tracts (CHT) districts bordering India and Myanmar (11%) [3].

Malaria prevention and control has gained increased momentum in the past decade [4]. Among the preventive measures, use of insecticide-treated bed nets (ITN)/long-lasting insecticide-treated nets (LLIN) is found to be an effective public health tool for control of malaria, especially among under-five children and pregnant women-the two most vulnerable groups [5-9]. This has been compared with generation of 'herd immunity' as in the case of vaccines [10]. But for this to happen, coverage has to be 'sufficiently high'. For a family size of five, three bed nets are recommended. To achieve this level of coverage (beyond 80%), mass distribution of insecticidal bed nets (ITN/LLINs) is recommended [10], including free distribution for equitable coverage [11]. However, the use of insecticidal bed nets is conditioned by knowledge on malaria transmission [12].

BRAC, an indigenous non-governmental development organization (NGO), had been implementing malaria prevention and control programme in the 13 malaria-endemic districts under the National Malaria Control Programme since 2007, with funding from GFATM. This is a collaborative effort of the Government of Bangladesh and a BRAC-led NGO consortium consisting of 20 smaller partner NGOs [13]. The overall goal of the programme is to reduce the burden of malaria in endemic areas by 2012. Free distribution of ITNs/LLINs is an important component of the programme, besides building awareness, and early diagnosis and prompt treatment of malaria following WHO guidelines.

A team of researchers from BRAC Research and Evaluation Division is engaged to support the programme through research on relevant issues of operation and effectiveness. As part of this, two cross-sectional surveys on the possession and usage of insecticidal bed nets were done in 2008 and 2011. In this paper data from these surveys were used to examine the progress made in the above aspects during the study period, and also the pro-poor inclination of the programme.

## Methods

Data for this study originated from two cross-sectional surveys done in June 2008 (pre-malaria dry season) and January-February 2011 (post-malaria dry season), one year and three-and-half years respectively after the programme began in July 2007. Both the surveys measured the possession and preferential use of insecticidal bed nets (for under-five children and pregnant women) by the households under the operation areas of BRAC-led NGO consortium. Data were compared between 2008 and 2011 in the south-eastern and north-eastern districts, and achievements checked against WHO recommended standards which stipulate "to ensure that at least 80% of those at risk of, or suffering from, malaria should benefit from major preventive and curative interventions by 2010" [7].

### The intervention

The LLINs are distributed to poor households by the community health workers (CHWs). They, with help from local office, also arrange camps to treat ordinary nets with insecticides. The CHWs are recruited from among the members of the women's credit and development groups of the respective NGOs through a participatory mechanism and receive training for 18 days in basic health care, monthly refreshers and orientation training on malaria [13]. They are the frontline workers of the NGOs' health programmes and act as a bridge between the community and government primary health care facilities. Each CHW is responsible for overseeing 75 households in the malaria-endemic areas and visits around 10 households in a day. During the visit she disseminates information on malaria and its transmission, educates on the norms of use of insecticidal bed nets (e.g., when to hang the net, where and how to wash and dry the net, and how many washes in a year etc.), and diagnose (by rapid diagnostic test, RDT) and treat malaria promptly following an algorithm. The RDT negative cases are referred to designated laboratory for microscopic examination and necessary management. Patients are referred to nearest health facilities without delay if children under five years or pregnant women show signs of complications. Information, Education and Communication (IEC) materials are used for awareness-building in interpersonal communication during household visits, health forums and informal discussions. Messages are also delivered through folk songs, popular theatre and print media such as leaflets, bill boards etc. and audiovisual media such as radio and television spots. To raise community awareness and remove various misbeliefs and stigma, IEC campaigns are organized for different stakeholders such as community and religious leaders, teachers, village doctors, medicine shop-keepers and private practitioners. All services are provided free of cost to the households. The interventions are implemented within the larger framework of BRAC Health Programme.

### Sampling

**Figure 1:**
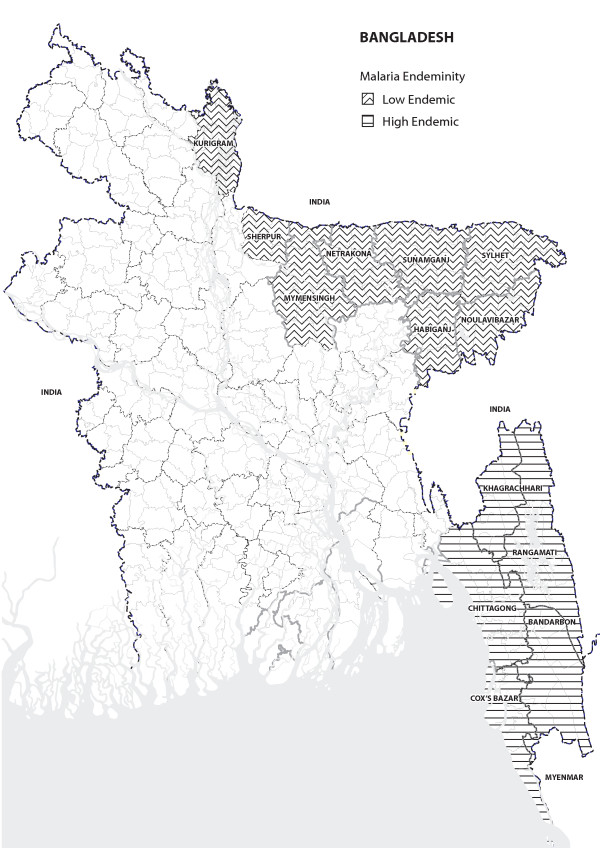
**Malaria endemic districts in Bangladesh**.

The principle behind the sampling strategy was that districts with higher prevalence of malaria would have more *upazilas *represented in the sample. This was done to accommodate programme length and depth resulting from its implementation strategy of beginning with the high endemic areas and gradually rolling over to the low endemic areas of the country. Due to constraints in time and resources, a convenient sample of 30 *upazila*s were selected randomly based on the above principle, out of a total 70 *upazila*s (in five high-endemic south-eastern districts and eight low endemic north-eastern districts, see Figure 1) where National Malaria Control Programme is being implemented. First, two *upazila*s were selected from each district (n = 26); next, an additional *upazila *was selected from each of the three high prevalence CHT districts (n = 3) (prevalence of malaria in these districts was 11% compared to 7% and 0.5% in the south-eastern and north-eastern districts respectively) [3], and finally, one extra *upazila *was selected from the highest prevalence Khagrachari district (prevalence 15.5%). From each of the selected *upazila*s, in 2008 one village and in 2011 two villages were chosen, the latter to reflect the greater number of villages covered in 2011. Thus, 30 villages in 2008 and 60 villages in 2011 (to accommodate scale of programme) were included in the surveys. The villages were chosen randomly from a list of villages where either LLINs had been distributed by the programme or a six-monthly retreatment programme of regular bed nets with insecticides (K-O tablets) was being implemented. Again, in a village, data were collected from HHs that had either one under-five child and/or a pregnant woman, each household being included only once in the survey. It may be mentioned here that no other agency had had distributed insecticidal bed nets in these villages earlier.

### The survey

Information was collected through face-to-face interview with respondents using a pre-tested structured questionnaire. The questionnaire was developed based on review of the literature on insecticidal bed nets and researchers experiences. It included questions on socio-demographics of the households, and possession and use of LLINs/ITNs. Data were collected from the household head or spouse, or a knowledgeable person in their absence. The survey team comprised of experienced interviewers and their supervisors. In hilly areas where an ethnic minority language was the primary language, interviewers from respective ethnic groups were recruited to conduct the interviews. A two-day intensive training organized for the interviewers consisted of didactic lectures, mock interviews, role play and field practice at community level. Several teams worked in parallel in each district. Households were visited on three repeated occasions at intervals if the first attempt was not successful. The field activities were supervised by the researchers who provided guidance as and when needed.

### Operational definitions

When individuals living together took meal from a common cooking facility, the entity is defined as a household (HH). Self-rated poverty status was measured by eliciting respondent's perception about the state of household's annual consumption expenditure in relation to income in the past year, which is documented to be a valid indicator of household stratification in rural Bangladesh [14]. Households were thus categorized as being in 'chronic deficit' (when running in deficit round-the-year or seasonally) or 'non-deficit' state. 'Insecticidal' bed net included both LLIN (distributed by the programme) and ITN (regular bed net which is retreated with insecticides six-monthly by the programme). Use of bed net was recorded if any household member(s) reportedly slept under an insecticidal bed net in the night before the day of survey. Pregnancy status was based on self-report only.

### Ethical approval

The study passed through the institutional review process at BRAC Research and Evaluation Division (Internal Review and Publication Committee) for ethical approval. Informed verbal consents (consent form read and explained) were obtained from the largely illiterate/semi-literate respondents. When the investigator was satisfied that the respondent understood it including its implications, and had agreed to participate, only then was s/he included in the survey. Anonymity of the respondents was maintained at all stages of data analysis.

### Data analysis

Data were analysed comparing two time periods (2008 and 2011) with respect to key outcomes of interest (such as HHs possessing at least a LLIN/ITN, HH members sleeping under an insecticidal bed net the night before, knowledge on norms of bed net use), separately for the two study areas (south-eastern and north-eastern districts). Achievements were cross-checked against WHO recommended 80% cut-offs where feasible. Pro-poor inclination of the programme was verified by analysis according to self-rated poverty status of the households. SPSS (version 11.5) was used for data analysis. Appropriate statistical test (e.g., *χ*^2 ^test) done where necessary, and 95% confidence interval (CI) calculated.

## Results

In 2008, the total households surveyed (with either a pregnant woman or an under-five child) was 3,760 (mean family size 5.4 ± 2.2) and in 2011 was 7,895 (mean family size 5.4 ± 2.1). Among the households surveyed, 94.4% in 2008 and 94.7% in 2011 had at least one under-five children. Also among these households, 14.7% in 2008 and 10.9% in 2011 had at least one pregnant woman. In 2008 and 2011, 21% and 30% of the household heads respectively had > 5 years of formal schooling. The majority of the household heads were engaged in wage employment (35% and 30% respectively in 2008 and 2011).

Table 1 shows information on bed nets possession. Both the proportion of households with at least one LLIN or at least one either of the LLIN or ITN increased over time, especially in the north-eastern districts. The mean number of insecticidal bed nets also increased in both areas during the study period and was more pronounced in the north-eastern districts.

**Table 1:** Households' (HHs) bed nets possession by study areas

	South-eastern districts		North-eastern districts		*χ*^2 ^value (95% CI)	
			
	2008	2011	2008	2011		
	
	a	b	c	d	a vs b	c vs d
% HHs with at least one bed net	90.2	97.9	90.5	95.9	160.7 (159.1-162.3)	73.7 (73.1-74.3)

% HHs with at least one LLIN	59.5	67.2	21.8	62.0	30.4 (30.2-30.6)	891.2 (879.2-903.3)

% HHs with at least one ITN	29.8	35.1	0.4	33.6	14.8 (14.7-14.9)	869.2 (857.6-881.1)

% HHs with at least one LLIN/ITN	64.3	76.0	21.9	74.4	79.1 (78.4-79.8)	1551.9 (1529.3-1574.8)

Mean No. of LLIN/ITN per household	1.0	1.83	0.2	1.69	---	---

N	1,701	3,724	2,059	4,171		

Greater than 70% of the household members in 2011 slept under an insecticidal bed net, a significant improvement from 2008 especially in the north-eastern districts (Table 2). There was also significant improvement in the proportion of pregnant women who slept under an insecticidal bed net in 2011 compared to 2008, in both areas.

**Table 2:** Utilization of LLINs/ITNs by household (HH) members by study areas

	South-eastern districts		North-eastern districts		*χ*^2 ^value (95% CI)	
			
	2008	2011	2008	2011		
	
	a	b	c	d	a vs b	c vs d
% HH members who slept under LLINs/ITNs the night before	75.8	77.2	58.6	71.8	1.4 (0.3-7.2)	109.4 (108.4-110.5)

N	5,849	15,210	2,390	17,395		

% under-five children who slept under LLIN/ITNs the night before	91.7	91.7	86.7	87.2	---	---

N	1,294	3352	578	3852		

% pregnant women who slept under LLINs/ITNs the night before	84.3	91.1	76.7	83.3	1,400.2 (1,380.2-1,420.6)	39.2 (38.9-39.5)

N	134	292	73	317		

Compared to the possession and prioritized use of insecticidal bed net for under-five children and pregnant women, compliance with its norms of use presented a bleak picture (Table 3). A very small proportion (< 5%) were knowledgeable about all norms of insecticidal bed net use. The level of knowledge regarding critical time of hanging the net increased over time, but failed to rise above 50% in 2011. Knowledge about washing the nets (e.g. place of washing and drying) was around 58% in 2011 in the north-eastern districts, but was between 33-39% in south-eastern districts.

**Table 3:** Households' (HHs) knowledge on norms of using insecticidal bed nets by study areas%

	South-eastern districts		North-eastern districts		*χ*^2 ^value (95% CI)	
			
	2008	2011	2008	2011		
	
	a	b	c	d	a vs b	c vs d
Norms of using LLINs/ITNs by HHs						

LLINs/ITNs to be hanged just before evening starts	21.9	54.1	7.2	43.7	491.3 (485.3-497.4)	848.8 (837.4-860.3)

Three/four washes in a year	30.6	28.3	12.0	35.6	3.0 (0.5-18.7)	5437.8 (5345.3-5532.0)

No wash directly in the pond or canal water	31.6	32.8	11.8	58.0	0.77 (0.1-7.8)	1202.8 (1185.9-1220.0)

Not to keep in the sun after washing	20.4	39.5	7.7	58.3	191.1 (189.2-193.2)	1450.8 (1429.9-1472.0)

Knows all norms of LLINs/ITNs use	5.3	1.6	1.8	3.7	52.2 (51.8-52.6)	16.5 (16.4-16.6)

N	1,701	3,724	2,059	4,171		

The pro-poor inclination of the programme is reflected in the status of relevant indicators according to self-rated poverty status of the households (Table 4). For all key indicators except children sleeping under insecticidal bed net (already surpassing the 80% cut-off), significant improvements were observed during 2008-2011 for the chronically deficit households. This again was, at par or marginally better, than that of the level for non-deficit households in 2011.

**Table 4:** Key insecticidal net- related indicators by household's self-rated poverty status

	Deficit households		Non-deficit households		*χ*^2 ^value (95% CI)	
			
	2008	2011	2008	2011		
	
	a	b	c	d	a vs b	c vs d
% HHs with at least one LLIN/ITN	43.3	75.9	37.6	73.2	548.6 (541.8-555.6)	738.1 (728.5-747.9)

% HH that hang LLINs/ITNs Just before evening	24.6	31.2	25.5	31.2	24.3 (24.2-24.5)	21.3 (21.2-21.4)

N	2,302	5,844	1,458	2,051		

% of HH members who slept under LLINs/ITNs the night before	70.1	74.7	72.0	73.3	12.7 (12.5-13.0)	1.2 (0.4-3.3)

N	5388	23,507	2856	9098		

% of under -five children who slept under LLINs/ITNs the night before	90.3	90.1	89.9	89.3	---	0.5 (0.0-2336.0)

N	1236	5423	636	1781		

% of pregnant woman who slept under LLINs/ITNs the night before	79.2	87.6	85.4	85.6	64.3 (63.8-64.9)	---

N	125	446	82	163		

Finally, the level of knowledge of the respondents after four years of intervention is shown in Table 5. Findings show a regional divide with the north-eastern districts lagging behind, and also the respondents still lagging far behind in knowledge on malaria, in both areas. These include, for example, critical knowledge on malaria such as transmission by 'bite of mosquito which has bitten a malaria patient' (varying from 22-36%) and its prevention 'using LLIN/ITNs' (varying from 28-38%) or by 'preventing breeding of mosquito' (varying from 9-15%). The majority of the respondents (55-82%) still perceived that using traditional bed net would prevent malaria while another 40-60% did not know how malaria is transmitted. However, majority of the respondents informed that they would go to allopathic provider/facilities for seeking treatment for malaria.

**Table 5:** Households' knowledge on causation, transmission, prevention and treatment of malaria in 2011 by study areas (multiple responses) (%)

	South-eastern districts	North-eastern districts	*χ*^2 ^value (95% CI))
Causes of malaria			

Mosquito bite	94.2	92.0	1.84(0.2-16.4)

Fly/insect bite	1.6	1.6	---

Lack of cleanliness	15.7	8.0	108.6 (107.6-109.6)

Don't know	1.6	3.9	39.4 (39.1-39.7)

Mode of transmission			

By bite of any mosquito	20.6	10.9	139.8 (138.5-141.2)

By bite of mosquito which has bitten a malaria patient	35.8	21.2	203.7 (201.6-205.9)

Other	0.1	0.0	0.8 (0.1-4.8)

Do not know	40.1	63.5	449.0 (443.6-454.5)

Mode of prevention			

Using LLIN/ITN	27.1	36.5	85.4 (84.6-86.1)

Preventing breeding of mosquito	15.0	8.5	81.9 (81.3-82.7)

Using traditional bed net	79.5	53.3	651.7 (643.3-660.2)

Using mosquito repellent/coil	8.9	4.8	51.8 (51.4-52.2)

Do not know	3.1	9.7	138.9 (137.6-140.3)

Possess knowledge about availability of free treatment of malaria	82.8	75.5	96.6 (95.8-97.5)

Place(s) of treatment			

CHW	57.8	56.9	0.6 (0.0-94.1)

Government Hospital	69.5	49.9	318.4 (314.8-322.1)

Non-government hospital/clinic	21.7	6.2	389.6 (385.0-394.3)

Village doctor	10.3	12.9	12.6 (12.3-12.9)

Drug shop attendants	5.5	2.7	39.2 (38.9-39.5)

Other(s)	1.2	7.1	163.6 (161.9-165.2)

N	3661	4065	

## Discussion

This study was done to assess the progress in the possession and usage of insecticidal bed nets under the National Malaria Control program in Bangladesh (NGO community component) implemented by an NGO-led consortium, and also, its avowed pro-poor inclination. Findings reveal a substantial improvement in possession and usage of insecticidal bed nets especially for the two most vulnerable groups (under-five children and pregnant women), and in reducing the gaps between the high endemic south-eastern districts and low endemic north-eastern districts during the study period. However, improvement in requisite knowledge for informed decision-making on prevention and treatment of malaria was not observed. The implications of these findings for future operation of the programme are discussed.

Compared to 2008, significant improvement was observed in 2011 regarding the distribution of LLINs (more pronounced in the north-eastern districts) as well as number of insecticidal bed nets possessed by the households. This is not surprising as it has been seen that free distribution is associated with rapid increase in ownership [11]. Though household possession of insecticidal nets is yet to match the optimum number suggested (e.g., three nets for a family of five), the distribution was found to be equitable favouring the chronic deficit households. This supports existing evidence that free distribution of insecticidal nets is more equitable in nature [10,15,16] compared to market-based distribution [17]. Programme needs to maintain this focus on equitable distribution so as to reverse the 'inverse care law' [18].

It was found that nearly 90% of the under-fives and the pregnant women were sleeping under an insecticidal net. This 'sufficiently high' coverage is a pre-requisite for tapping the 'herd immunity' effect from insecticidal nets [10]. This happens in two ways: the spill-over effect of hanging an insecticidal net in the vicinity [19], and reduction of reservoir of infection through reduction of vector life-span [20]. Encouragingly, households which possessed insecticidal nets used it preferentially for the vulnerable groups mentioned above.

The commendable performance in insecticidal bed net possession and use was not matched by the level of knowledge on transmission, prevention and treatment of malaria among the respondents even four years after the beginning of the programme, and remained superficial. This was especially noticeable regarding knowledge on norms of using and maintaining insecticidal nets. For promoting proper use of insecticidal nets (e.g., critical time of hanging, mode of washing and drying etc.), improvement in knowledge on malaria transmission and prevention is essential [12]. Dissemination of comprehensive information on different aspects of malaria will help in informed decision-making by the community and will expedite uptake of preventive and curative services offered by the programme. Also, this information should be culture-sensitive to be acceptable to the community [21].

During the study period, the regional divide in different indicators of insecticidal bed nets decreased as also the difference between the deficit and non-deficit households which shows pro-poor inclination of the programme. This is understandable because the programme's initial thrust was in the high endemic south-eastern districts and gradually rolled over to the rest. The programme need to maintain this focus in future.

This study was carried out in malaria off seasons and it is possible that carrying out the study in peak seasons would have altered the findings e.g., with respect to use by the vulnerable groups due to rainfall and humidity. Sampling was done on the basis of level of malaria prevalence in the districts and might have biased towards the high-endemic areas e.g., CHT districts. The findings are based on self-reported data and not direct observation and thus, under or over-reporting could not be ruled out especially since net usage has been shown to be consistently lower in the dry seasons [22]. As it is a cross-sectional survey, direct attribution to programme effects cannot be made.

## Conclusions

The programme is on right track with respect to equitable distribution of the insecticidal bed nets and its preferential use by the vulnerable groups such as the under-five children and the pregnant women. However, challenges remain regarding improvement of critical knowledge on malaria for informed decision-making for prevention and treatment. Finally, the "catch-up (mass, free distribution)" campaigns should be backed by "keep-up (long-term, routine access to new nets)" process to maintain high coverage over time [23], and sustain the achievements already made.

## Competing interests

The authors declare that they have no competing interests.

## Authors' contributions

SMA conceptualized and designed the study. Analysed data and interpreted results. Wrote the first draft and subsequently revised it for submission. SH participated in the design and sampling, field management, and analysis of data. MA took part in interpretation of the data and contributed in drafting and revising the manuscript. SR participated in design, analysis and interpretation of data including critical revision of the manuscript. All authors read the final manuscript and approved for submission.
